# Prognostic Factors for COVID-19 Pneumonia Progression to Severe Symptoms Based on Earlier Clinical Features: A Retrospective Analysis

**DOI:** 10.3389/fmed.2020.557453

**Published:** 2020-10-05

**Authors:** Huang Huang, Shuijiang Cai, Yueping Li, Youxia Li, Yinqiang Fan, Linghua Li, Chunliang Lei, Xiaoping Tang, Fengyu Hu, Feng Li, Xilong Deng

**Affiliations:** Guangzhou Eighth People's Hospital, Guangzhou Medical University, Guangzhou, China

**Keywords:** COVID-19, SARS-CoV-2, risk factor, clinical manifestation, prognostic factor

## Abstract

Approximately 15–20% of COVID-19 patients will develop severe pneumonia, and about 10% of these will die if not properly managed. Earlier discrimination of potentially severe patients basing on routine clinical and laboratory changes and commencement of prophylactical management will not only save lives but also mitigate the otherwise overwhelming healthcare burden. In this retrospective investigation, the clinical and laboratory features were collected from 125 COVID-19 patients who were classified into mild (93 cases) or severe (32 cases) groups according to their clinical outcomes after 3–7 days post-admission. The subsequent analysis with single-factor and multivariate logistic regression methods indicated that 17 factors on admission differed significantly between mild and severe groups but that only comorbidity with underlying diseases, increased respiratory rate (>24/min), elevated C-reactive protein (CRP >10 mg/L), and lactate dehydrogenase (LDH >250 U/L) were independently associated with the later disease development. Finally, we evaluated their prognostic values with receiver operating characteristic curve (ROC) analysis and found that the above four factors could not confidently predict the occurrence of severe pneumonia individually, though a combination of fast respiratory rate and elevated LDH significantly increased the predictive confidence (AUC = 0.944, sensitivity = 0.941, and specificity = 0.902). A combination consisting of three or four factors could further increase the prognostic value. Additionally, measurable serum viral RNA post-admission independently predicted the severe illness occurrence. In conclusion, a combination of general clinical characteristics and laboratory tests could provide a highly confident prognostic value for identifying potentially severe COVID-19 pneumonia patients.

## Background

The novel coronavirus (SARS-CoV-2) has seemed to sweep across the globe ever since its first successful jump from bat to human being through a still unknown intermediate(s) in approximately late Nov 2019; it still shows a tendency toward significant surges in incidence worldwide (1–3). The SARS-CoV-2 virus seems more contagious than its sibling virus, severe acute respiratory syndrome (SARS) virus, which broke out in 2003; as of March 11, over 120,000 individuals have contracted COVID-19 pneumonia within 3 months, which was about 15 times that of the total SARS cases (8,000 in 7 months) (4). The surging increase in COVID-19 patients within a short time window will severely impact the limited medical resources, including physicians, nurses, protective suits, masks, and goggles. Data from the Chinese mainland showed that the majority of total infected patients will recover under simple supervision management, such as quarantine in a compartment hospital isolation ward, but that the overall case fatality rate was 2.3% (5). For the clinical treatment of COVID-19 patients under shortage of enough medical supplies, the critical issues and priorities are to treat the severe COVID-19 patients [about 20% of the whole population (5)] and to save their lives with preventive and intensive medical care. However, the clinical presentation of COVID-19 patients differs substantially, and this can include asymptomatic infection, mild upper respiratory tract illness, and severe viral pneumonia (2, 6–8). The most crucial issue is therefore to identify these patients and prioritize their treatment strategy by applying prophylactic medical treatment and management before they progress to the severe stage.

As we know, respiratory function worsens in the severe stage. In clinical practice, saturated oxygen (<93% in rest state), reparatory rate (>30 times/min), and deteriorated chest radiology imaging (X-Ray and CT more high resolution) provide references to confirm their severity (5, 9, 10). Because of the hypoxia stress, most patients will experience an over-reactivated immune storm, including elevated expression levels of some specific immunological cytokines and changes in certain types of immune cell counts (6, 11). Biopsy analysis also showed that the lung bilateral diffuse alveolar damage with cellular fibromyxoid exudates (12). However, CT imaging and immunology detection is not only expensive but also largely unavailable an unable to cope with the significant rise in suspected cases, particularly in those hospitals that are not well-equipped. Can some routine clinical characteristics or/and laboratory measurements (or their combination) predict the occurrence of severe cases?

In this study, we retrospectively analyzed the clinical characteristics of those patients who progressed to severe pneumonia later and found that five simple clinical features and laboratory detection at an earlier time point could serve as prognostic factors facilitating discrimination of severe cases in advance.

## Methods

### Patients

COVID-19 diagnosis was determined according to the criteria in the new Coronavirus pneumonia diagnosis and treatment plan (trial version 6) issued by the National Health and Health Commission (13). All 298 COVID-19 patients admitted to Guangzhou Eighth People's Hospital from January 20 to February 29, 2020, were included in this study. This study complied with the medical ethics of Guangzhou Eighth People's Hospital. We obtained written consent from the patients.

For this analysis, inclusion criteria were the following: (1) diagnosed as mild or ordinary on admission and (2) length of hospitalization >3 days and overall duration of the disease >7 days. Qualified patients were then classified into a mild symptom group and a severe symptom group based on the clinical manifestation. The severe symptom diagnosis was determined according to the following criteria: (1) respiratory distress, RR ≥ 30 times/min in the resting state; (2) oxygen saturation ≤ 93% in the resting state; and (3) arterial blood oxygen partial pressure (PaO_2_)/oxygen concentration (FiO_2_) ≤ 300 mmHg). The rest of the patients were in the mild group.

### Data Collection

Patient general information, including gender, age, underlying diseases, epidemic history, etc., and their clinical data including symptoms, signs, clinical classification (course duration >7 days), laboratory test results, and SARS-CoV-2 viral test results were obtained with standardized data collection forms from electronic medical records.

### Statistical Analysis

Quantitative data was firstly tested to be normality distribution with the Kolmogorov-Smirnov method. Then, for normalized distributed data, *t*-test and Tamhane T2 methods were used for variance of even and uneven data, respectively. For non-normal data, which was expressed as the median (quartile) [M (P25, P75)], the Mann-Whitney *U*-test was employed. The chi-square test (or Fisher exact probability method) was utilized for analyzing qualitative data. Logistic regression analysis and the receiver operating characteristic curve (ROC) analysis was employed to analyze the independent risk factors. The difference was statistically significant at *P* < 0.05. All analysis was performed using SPSS software (version 20.0).

## Results

### Patient General Information

**Figure 1:**
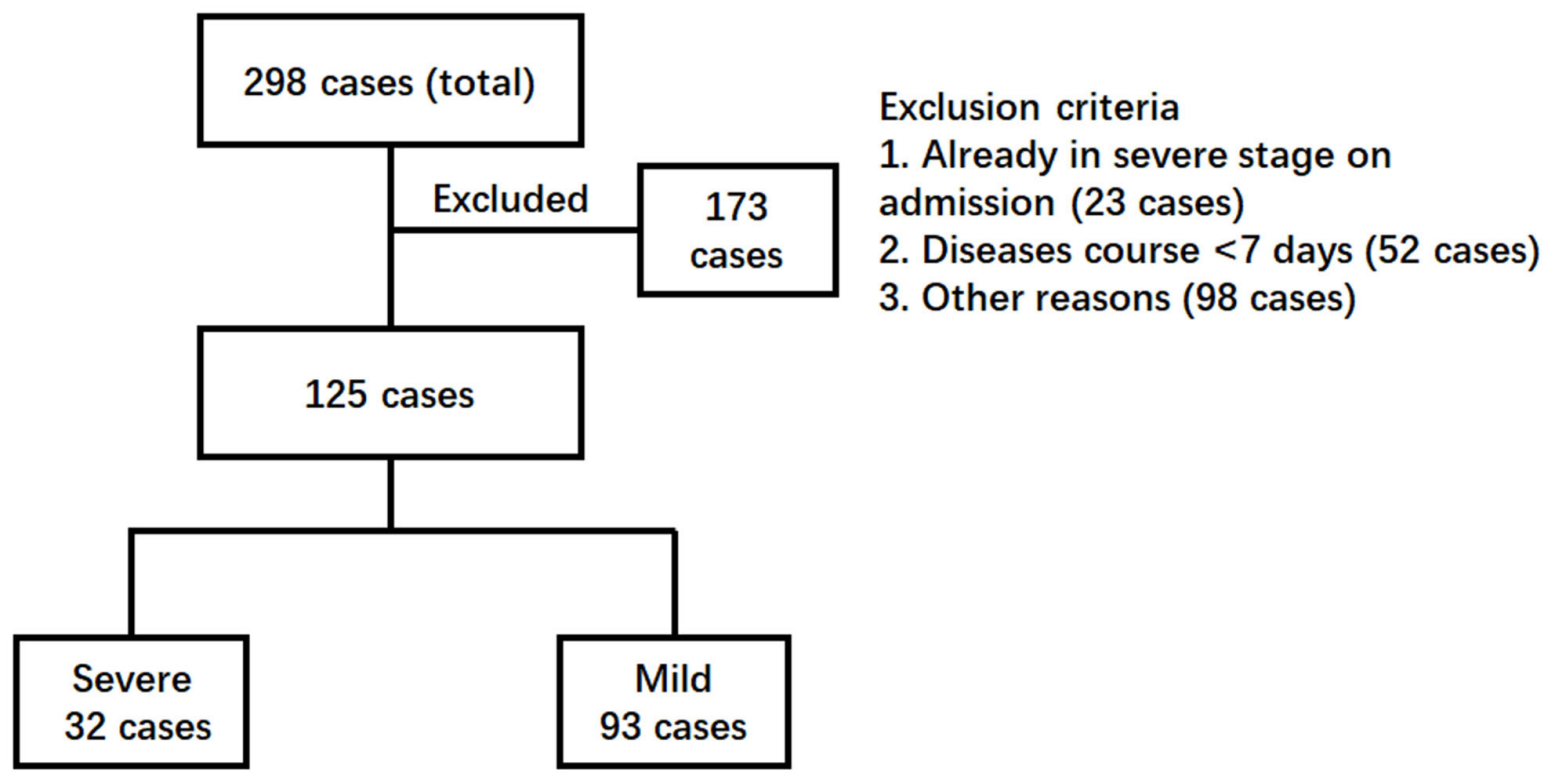
Enrollment chart of COVID-19 patients. The clinical information of a total of 298 patients admitted to the hospital was reviewed. Patients were excluded according to the criteria (1) already in severe stages, (2) with a disease course <7 days, and (3) other reasons such as incomplete detection panel. Finally, 125 patients were further divided into two groups. The severe group included the patients who developed severe COVID-19 pneumonia later (>3 days post-admission). The patients remaining were kept in the mild group.

A total of 298 COVID-19 cases (about 85% of total cases in Guangzhou, China) were admitted to Guangzhou Eighth People's Hospital for treatment from January 20 to February 29, 2020 (Figure 1). According to the inclusion criteria, 173 cases were excluded because 23 cases were already in the severe symptom stage, 52 cases had a short hospitalization time of <7 days, and 98 patients had other defects, such as being short of a complete set of detection. Finally, 125 cases, including 63 males and 62 females, were qualified to be included for further investigation, and all their disease courses were over 7 days, with a maximum of 32 days. Based on the severity of disease at 3 days post-admission, 93 patients fell in the mild group (38 general cases and 55 mild cases) and 32 patients in the severe group (25 severe cases and seven critical cases).

All included patients were aged between 1.5 and 91 years (averaged 44.87 ± 18.55 years) (Table 1). Among them, 37 cases had at least one underlying disease, including 20 cases with hypertension, eight cases with diabetes, five cases with coronary heart disease, two cases with chronic obstructive pulmonary disease, two cases with chronic kidney disease, two cases with chronic liver disease, and two cases with sleep apnea syndrome. Five individuals with two or more basic disorders and 7 cases with obesity (BMI > 26). Epidemiologically, 88 cases had a history of traveling to or living in the Hubei epidemic area before disease onset. Interestingly, we observed that seven patients developed serum SARS-CoV-2 viral RNA positive after admission but ahead of diagnosis to be a severe symptom.

**Table 1:** Characteristics of COVID-19 patients.

	**All cases**	**Mild (*n* = 93)**	**Severe (*n* = 32)**	***p*-value**
Gender				0.96
Male	63	47	16	
Female	62	46	16	
Age (years)^***^	44.87 ± 18.55	40.49 ± 17.66	59.43 ± 13.47	<0.05
Underlying disease (cases)	37	17	20	<0.05
Hypertension (cases)	20	7	13	<0.05^*^
Diabetes (cases)	8	2	6	<0.05^*^
Obesity (BMI>30) (cases)	7	2	5	<0.05^*^
Travel to epidemic area (cases)	88	65	23	0.388
Temperature (cases)				<0.05
<37.4°C	57	48	9	
37.4–38.5°C	48	34	14	
>38.5°C	20	11	9	
Coughing (cases)	76	56	20	0.819
Running nose (cases)	21	14	7	0.533
Muscle joint pain (cases)	27	23	4	0.147
Headache (cases)	24	16	8	0.334
Fatigue (cases)	48	32	16	0.061
Digestive Symptoms (cases)	19	15	4	0.622
Fast respiratory rate (cases)	20	4	16	<0.05^*^
Serum viral RNA positive (cases)	7	0	7	<0.05^*^
White cell counts (10E9/L)^***^	5.57 ± 1.76	5.65 ± 1.73	5.33 ± 1.86	0.411
Absolute neutrophil counts (10E9/L)^***^	3.43 ± 1.43	3.26 ± 1.28	34.97 ± 1.77	0.053
Absolute leukocyte counts (10E9/L)^&^	1.32 (1.05–2.18)	1.43 (1.23–2.21)	0.82 (0.57–1.05)	<0.05^**^
Absolute eosinophil counts (10E9/L)^&^	0.02 (0–0.09)	0.04 (0.1–0.12)	0(0–0)	<0.05^**^
Platelets (10E9/L)^***^	200.56 ± 56.24	206.01 ± 55.61	182.46 ± 55.47	0.052
Hemoglobin (g/L)^***^	134.39 ± 18.02	135.31 ± 17.92	131.32 ± 18.32	0.306
Prothrombin time (sec)^***^	13.69 ± 1.13	13.66 ± 0.89	13.80 ± 1.70	0.668
Activated partial prothrombin time (sec)^***^	39.30 ± 4.74	38.93 ± 4.49	40.49 ± 5.37	0.13
C-reactive protein (CRP) (mg/L)^&^	6.32 (1.63–23.50)	4.00 (1.06–12.41)	46.345 (28.97–60.50)	<0.05^**^
D-dimer (μg/L)^&^	910 (700–1,400)	780 (560–1,050)	1,760(1297.5–3,265)	<0.05^**^
Procalcitonin (ng/ml)^&^	0.047 (0.03–0.076)	0.037 (0.027–0.063)	0.070 (0.051–0.145)	<0.05^**^
Lactic acid (mmol/L)^***^	1.78 ± 0.71	1.72 ± 0.76	1.93 ± 0.55	0.207
Alanine aminotransferase (ALT, U/L)^&^	18.90 (13.40–25.20)	16.70 (12.40–22.15)	27.5 (19.70–41.25)	<0.05^**^
Aspartate aminotransferase (AST, U/L)^&^	18.40 (14.20–27.15)	17.20 (13.75–21.00)	31.50 (23.25–37.75)	<0.05^**^
Albumin (g/L)^***^	38.30 ± 5.30	39.83 ± 4.49	33.49 ± 4.79	<0.05
Creatinine (umol/L)^***^	67.15 ± 28.21	64.02 ± 26.95	77.56 ± 42.19	0.271
Creatine kinase (CK, U/L)^***^	82.89 ± 48.39	77.93 ± 46.05	100.27 ± 59.73	0.08
Lactate dehydrogenase (LDH, U/L)^&^	175 (150–241.5)	161 (145–192)	322 (279.75–400)	<0.05^**^

* Fisher's Exact Test.

** *Mann-Whitney U-Test*.

*** average ± standard deviation (STD).

& *verage (95% confidence interval)*.

### Factors Differed Between the Mild Group and the Severe Group

The single-factor analysis was applied for each factor between the mild group and the severe group (Table 1). More patients in the severe group were old, obese (BMI > 26), and had underlying diseases, particularly hypertension and diabetes (*P* < 0.05), compared with the mild group. Among the general factors, no significant difference could be seen with regards to gender, history of traveling to or living in an epidemic region, coughing, sneezing, muscle joint pain, headache, fatigue, and gastrointestinal symptoms between these two groups (*P* > 0.05). However, more patients in the severe group exhibited high fever, chest tightness, and shortness of breath (fast respiratory rate) (*P* < 0.05). The serum concentration of C-reactive protein, procalcitonin, D-dimer, albumin, and lactate dehydrogenase (LDH) increased significantly in the severe group (*P* < 0.05).

Compared to the mild group, patients in the severe group had lower absolute lymphocyte counts, higher eosinophil counts (*P* < 0.05), and similar levels of other parameters, including white blood cells, neutrophils, platelets, hemoglobin, prothrombin time, activated partial thromboplastin time, blood lactic acid, blood creatinine, and creatine kinase. Interestingly, the levels of glutamate aminotransferase (ALT) and aspartate aminotransferase (AST) significantly increased for severe patients (*P* < 0.05). However, the median values of ALT and AST were still within the normal range, indicating that most of the severe COVID-19 patients had no significant liver damage.

Importantly, all seven patients with the presence of SARS-CoV-2 viral RNA in blood during the hospitalization, but before being in the severe stage, finally progressed to the severe stage; they included two severe cases and five critical cases (*P* < 0.05).

### Binary Logistic Regression Analysis of COVID-19 Severe Risk Factors

Next, all categorical variables were converted into covariates, including age, presence of underlying diseases (Yes or No), hypertension (Yes or No), diabetes (Yes or No), obesity (Yes or No), Temperature (<37.4, 37.4–38.5, >38.5°C), fast respiratory rate (Yes or No), elevated C-reactive protein (>10 mg/L), decreased lymphocyte count (<1.1^*^10E9/L) and eosinophil count (<0.02^*^10E9/L), elevated procalcitonin (>0.05 ng/L), elevated D-dimer (>=2.25 μg/L), decreased albumin (<35 g/L), and elevated lactate dehydrogenase (LDH, >250 U/L), and were then subjected to single-factor logistic regression together with multiple independent variables. Those variables with statistical significance were chosen for subsequent binary logistic regression analysis to test the model coefficients, goodness-of-fit, and multicollinearity. Four factors identified to be significantly relevant to the severity of COVID-19 were underlying diseases (X1), fast respiratory rate (>24 times/min) (X2), elevated C-reactive protein level (CRP > 10 mg/L) (X3), and elevated lactate dehydrogenase level (LDH > 250 U/L) (X4) (Table 2). Finally, the multifactor logistic regression equation was obtained: *P* = −6.488 + 2.752X1 + 4.056X2 + 2.424X3 + 5.392X4. The β values and odds ratios (OR) for each factor are shown in Table 2. The result indicated that elevated LDH ranks as having the highest correlation to severe symptom development (OR = 219.608), followed by the fast respiratory rate (OR = 57.726), underlying diseases (OR = 15.67), and elevated CRP (OR = 11.289).

**Table 2:** Independent factors associated with severe symptom development in COVID-19 patients.

**Variables**	**β**	**S.E**.	**chi-square**	***P*-value**	**OR (95% confidence interval)**	
*X*_1_	Underlying disease	2.752	1.066	6.666	0.01	15.67 (1.94–126.55)
*X*_2_	Fast respiratory rate (>24 times/min)	4.056	1.183	11.76	0.001	57.726 (5.685–586.191)
*X*_3_	CPR (>10 mg/L)	2.424	1.004	5.823	0.016	11.289 (1.577–80.838)
*X*_4_	LDH (>250 U/L)	5.392	1.24	18.911	<0.001	219.608 (19.332–2494.742)
Intercept		−6.488	1.499	18.738	<0.001	0.002

S.E., standard error;

*SOR (95% CI), Odd Ratio (95% confidence interval)*.

### The Prognostic Capacity for Severe Symptom Development

To better evaluate the prediction capacity of each of the independent risk factors, we plotted their receiver operating characteristic curve (ROC) for the development of severe COVID-19 pneumonia and calculated the area under the ROC curve (AUC value), sensitivity, specificity, Cut-off value, Youden index, and *p*-value (Table 3) for all of them. According to the general standard that AUC values between 0.7 and 0.9 mean a medium level of diagnostic values and AUC values over 0.9 mean a high level of diagnostic values, we observed that all the factors (AUC <0.9) failed to provide a high prognostic value when used alone. A two-factor combination test then showed that the combination of fast respiratory rate and elevated LDH could provide a highly confident prediction (AUC = 0.944, sensitivity = 0.941, and specificity = 0.902) (Table 3). The AUC values of elevated LDH plus underlying diseases or plus elevated CRP were both over 0.9, but their sensitivity or specificity was lower than 0.9. Then, triple factor combination significantly increased the prognostic efficacy, and all combinations had increased sensitivity and specificity (Table 3). Finally, we calculated the prognostic value of the combination of all four factors and found that the AUC value was significantly increased to 0.985 (95% CI 0.968–1.000), the sensitivity to 0.912, and the specificity to 0.957 (Table 3).

**Table 3:** Prognostic values for severe COVID-19 pneumonia development.

	**Factor**	**AUC (95% CI)**	**Sensitivity**	**Specificity**	**Cut-off value**	**Youden Index**	***P-*value**
Single factor	Underlying diseases (1)	0.722 (0.614–0.829)	0.618	0.826	0.367	0.444	<0.001
	Fast respiratory rate (2)	0.758 (0.648–0.867)	0.559	0.957	0.492	0.516	<0.001
	Elevated CRP (3)	0.774 (0.685–0.864)	0.853	0.696	0.298	0.549	<0.001
	Elevated LDH (4)	0.855 (0.766–0.944)	0.765	0.946	0.461	0.711	<0.001
Two factors	(1) + (2)	0.853 (0.767–0.939)	0.824	0.793	0.223	0.617	<0.001
	(1) + (3)	0.854 (0.779–0.928)	0.853	0.696	0.274	0.549	<0.001
	(1) + (4)	0.940 (0.894–0.987)	0.971	0.783	0.156	0.754	<0.001
	(2) + (3)	0.870 (0.795–0.944)	0.912	0.663	0.18	0.575	<0.001
	(2) + (4)	0.944 (0.892–0.996)	0.941	0.902	0.315	0.843	<0.001
	(3) + (4)	0.918 (0.856–0.981)	0.765	0.946	0.365	0.711	<0.001
Three factors	(1) + (2) + (3)	0.910 (0.850–0.969)	0.765	0.902	0.253	0.667	<0.001
	(1) + (2) + (4)	0.976 (0.955–0.998)	0.912	0.935	0.411	0.846	<0.001
	(1) + (3) + (4)	0.963 (0.933–0.993)	0.912	0.891	0.227	0.803	<0.001
	(2) + (3) + (4)	0.964 (0.919–1.000)	0.912	0.934	0.355	0.847	<0.001
Four factors	(1) + (2) + (3) + (4)	0.985 (0.968–1.000)	0.912	0.957	0.374	0.869	<0.001

*AUC (95% CI), Area under receiver operating characteristic curve (95% confidence interval). The highlighted combinations are those with AUC > 0.900 plus sensitivity > 0.900 plus specificity > 0.900, which indicates significant and reliable prognostic values for clinical practice*.

## Discussion

Our study showed that underlying disease, fast respiratory rate (>24 times/min), elevated serum C-reactive protein level (CRP, >10 mg/L), and elevated lactate dehydrogenase level (LDH, >250 U/L) were four independent risk factors for predicting the progression of some COVID-19 patients from mild to severe conditions. Firstly, elevated lactate dehydrogenase levels ranked as number 1 (OR = 219.332) and fast respiratory rate as number 2 (OR = 57.726) among the four factors (Table 2). Interestingly, an elevated lactate dehydrogenase level was associated with severe SARS infection (14), which broke out in 2003, but was absent in the severe MERS infection (15), which is still circulating. When used individually, all four factors have a moderate prediction value for their low specificity and sensitivity (AUC values <0.9) (Table 3). Secondly, we found that the combination of two factors, fast respiratory rate plus elevated LDH, could provide a high prognostic value for severe symptom development (AUC = 0.944, sensitivity = 0.941, and specificity = 0.902). Combinations of triple factors could significantly increase the prognostic value (AUC > 0.9). Finally, a combination of all four factors, provide an excellent prognostic efficacy, achieving AUC = 0.985 (95% CI 0.968–1.000) with high sensitivity (0.953) and specificity (0.968).

Our hospital has treated over 80% of COVID-19 patients in Guangzhou city-−298 cases as of February 29, 2020—including 55 severe cases but only one death case. All the patients except two patients recovered as of March 15. A retrospective analysis of all the cases revealed that the extremely low fatality rate in our hospital, one of 298 cases (0.0336%)—significantly lower than the overall fatality rate (2.3%) in China (5), was largely attributed to the effect of an expert panel, consisting of physicians from multiple disciplines, including infectious diseases, respiratory diseases, and intensive care unit (ICU), and radiology. Patients newly admitted were reviewed by the panel, and patients who meet several of the following criteria were transferred immediately to the ICU isolation ward for close supervision, including, (1) the illness onset has entered 7–10 days; (2) over 50 years old; (3) obesity, pregnant women, children; (4) with underlying diseases, especially hypertension, diabetes, COPD; (5) fast respiratory rate; (6) obvious decline in spirit and appetite; (7) significant reduction and/or progressive decline of peripheral blood lymphocytes; (8) decrease in albumin; (9) elevated C-reactive protein; (10) elevated lactate dehydrogenase; and (11) quickly deteriorated or with two or more lesions in lungs revealed by chest imaging. Once they progressed to the severe stage, they received treatment immediately. The above four prognostic factors, as routine and affordable clinical characteristics, were included in these criteria, and their immediate and preventive therapies were facilitated retrospectively.

All seven patients who were detected to be serum viral RNA positive developed severe symptoms very soon, which further confirmed our previous observation that detectable 2019-nCoV viral RNA in blood is a reliable indicator for further clinical severity (16). However, as the viral RNA positive rate was low high (seven of 32 cases, 21.8%) in this study and other reports (17) and viral RNA detection is expensive, we do not recommend the continuous detection of viral RNA. In this regard, we suggest reserving the precious reagent for confirming virus infection.

In conclusion, our study indicated that underlying disease, a fast respiratory rate, elevated serum C-reactive protein level, and elevated lactate dehydrogenase level significantly correlated to the development of severe COVID-19 pneumonia; additionally, elevated lactate dehydrogenase and a fast respiratory rate (possibly plus one or two more other factors) can serve as prognostic factors for the discriminating potential severe cases among the mild COVID-19 patients. Our study provided convenient, reliable, and affordable references for both patients and physicians to make a highly confident decision to commence management and treatment safely.

## Summary

With our successful experience of treating COVID-19 patients, we retrospectively found that routine clinical features could reliably predict severe pneumonia development and could thus provide quick and affordable references for physicians to save patients with otherwise fatal COVID-19 using their limited medical resource.

## Data Availability Statement

The original contributions presented in the study are included in the article/supplementary material, further inquiries can be directed to the corresponding authors.

## Ethics Statement

Ethical review and approval was not required for the study on human participants in accordance with the local legislation and institutional requirements. The patients/participants provided their written informed consent to participate in this study.

## Author Contributions

HH, FL, and XD conceived the study and wrote the manuscript. HH and SC collected data and performed the data analysis. HH, SC, YuL, YoL, YF, and XD participated in the clinical treatment. LL, CL, and XT supervised the clinical treatment. FH analyzed the results. All authors read the manuscript and approved the final version. All authors contributed to the article and approved the submitted version.

## Conflict of Interest

The authors declare that the research was conducted in the absence of any commercial or financial relationships that could be construed as a potential conflict of interest.
